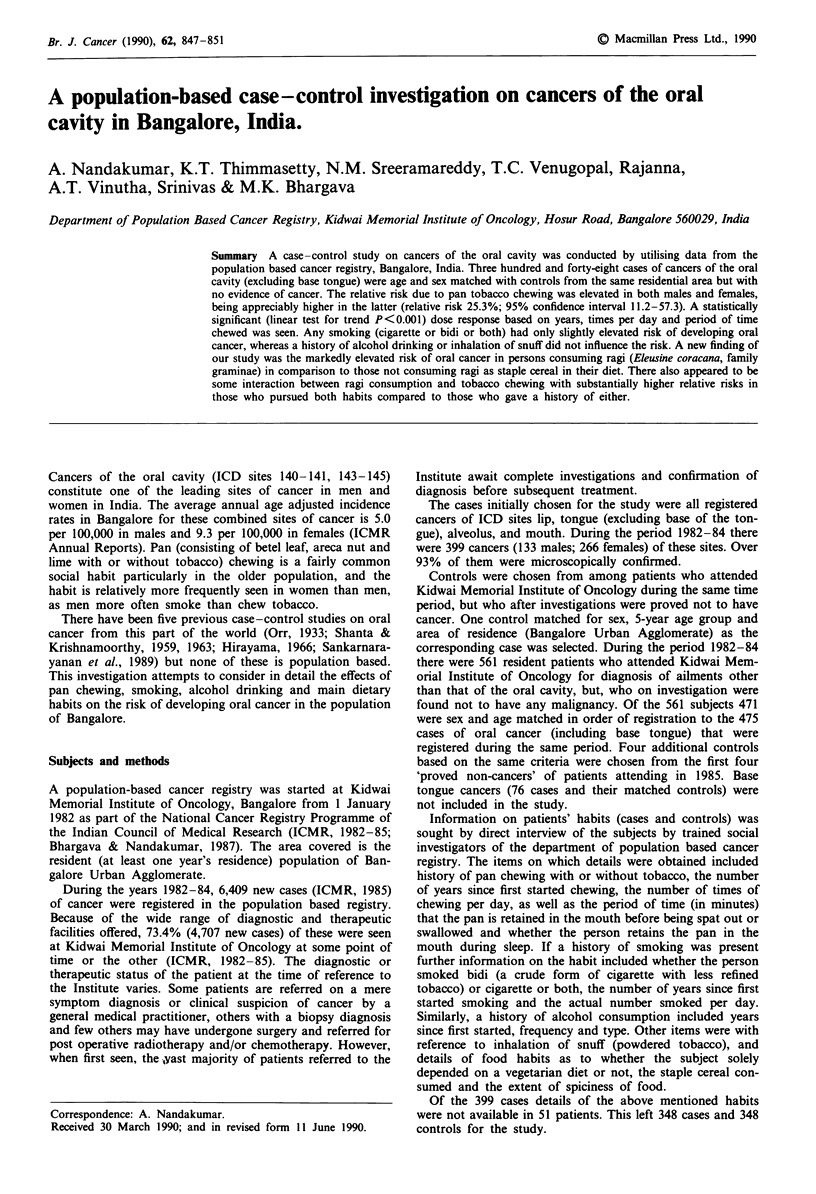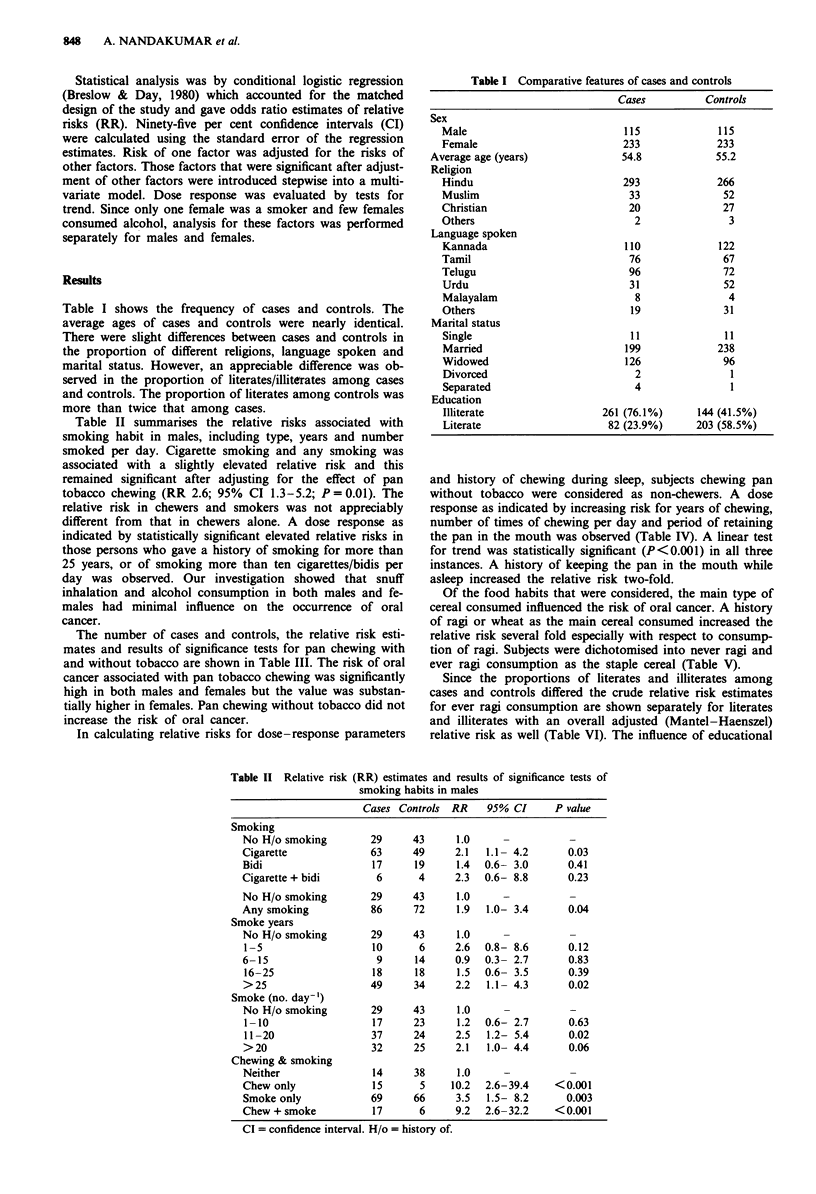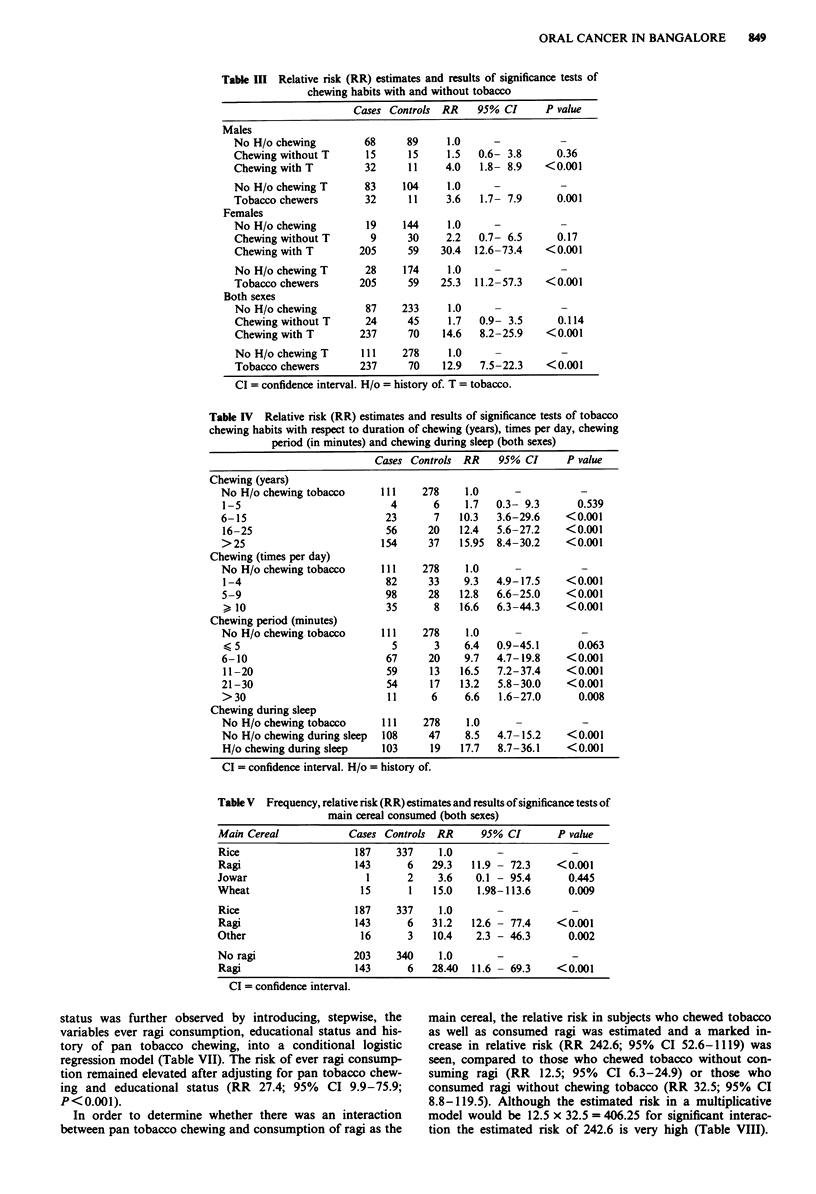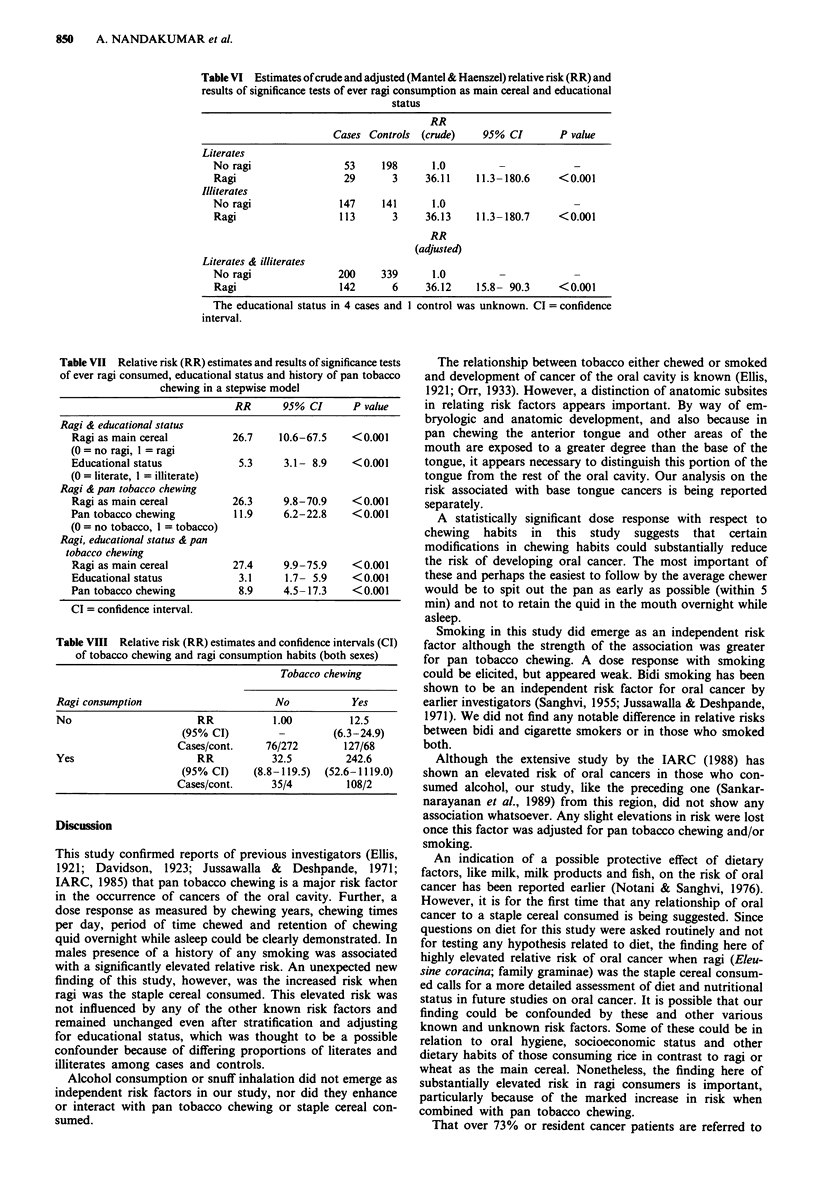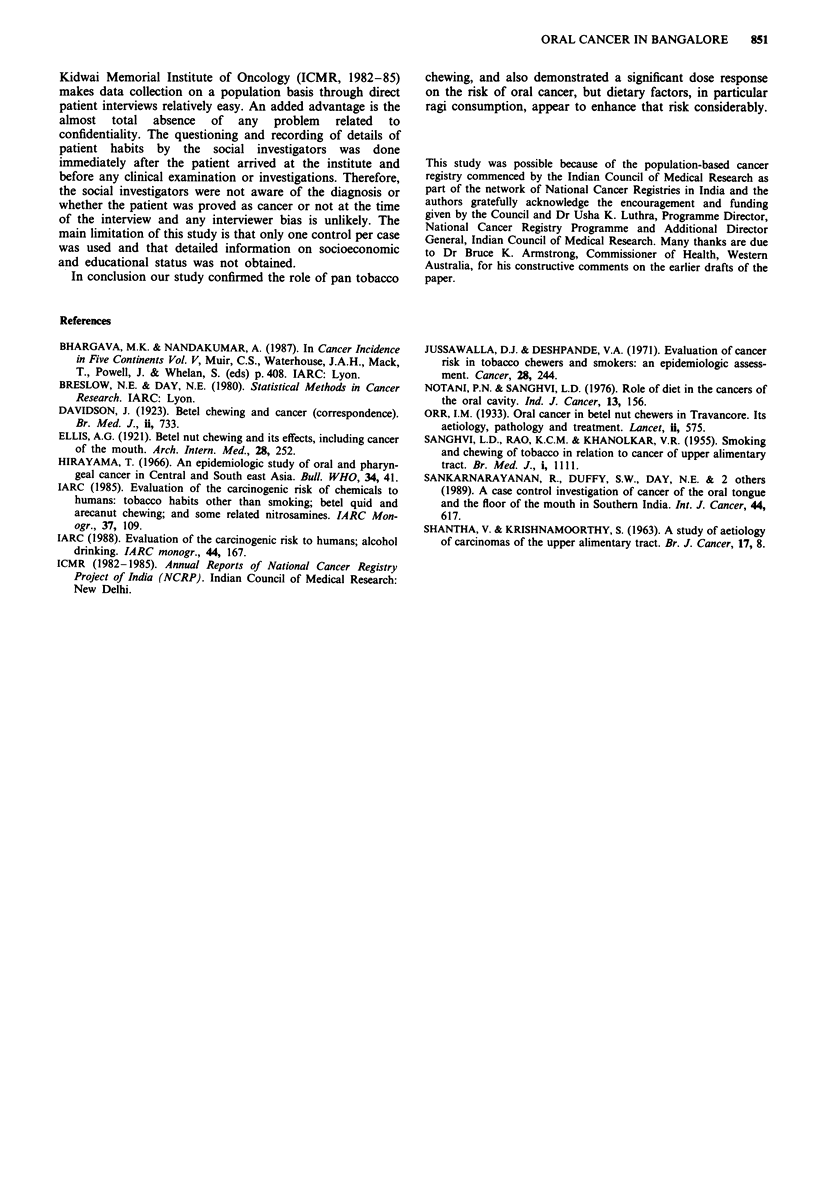# A population-based case-control investigation on cancers of the oral cavity in Bangalore, India.

**DOI:** 10.1038/bjc.1990.392

**Published:** 1990-11

**Authors:** A. Nandakumar, K. T. Thimmasetty, N. M. Sreeramareddy, T. C. Venugopal, A. T. Vinutha, M. K. Bhargava

**Affiliations:** Department of Population Based Cancer Registry, Kidwai Memorial Institute of Oncology, Bangalore, India.

## Abstract

A case-control study on cancers of the oral cavity was conducted by utilising data from the population based cancer registry. Bangalore, India. Three hundred and forty-eight cases of cancers of the oral cavity (excluding base tongue) were age and sex matched with controls from the same residential area but with no evidence of cancer. The relative risk due to pan tobacco chewing was elevated in both males and females, being appreciably higher in the latter (relative risk 25.3%; 95% confidence interval 11.2-57.3). A statistically significant (linear test for trend P less than 0.001) dose response based on years, times per day and period of time chewed was seen. Any smoking (cigarette or bidi or both) had only slightly elevated risk of developing oral cancer, whereas a history of alcohol drinking or inhalation of snuff did not influence the risk. A new finding of our study was the markedly elevated risk of oral cancer in persons consuming ragi (Eleusine coracana, family graminae) in comparison to those not consuming ragi as staple cereal in their diet. There also appeared to be some interaction between ragi consumption and tobacco chewing with substantially higher relative risks in those who pursued both habits compared to those who gave a history of either.


Br. J. Cancer (1990), 62, 847-851 © Macmillan Press Ltd., 1990

### A population-based case-control investigation on cancers of the oral cavity in Bangalore, India.

A. Nandakumar, K.T. Thimmasetty, N.M. Sreeramareddy, T.C. Venugopal, Rajanna, A.T. Vinutha, Srinivas & M.K. Bhargava

*Department of Population Based Cancer Registry, Kidwai Memorial Institute of Oncology, Hosur Road, Bangalore 560029, India*

**Summary** A case-control study on cancers of the oral cavity was conducted by utilising data from the population based cancer registry, Bangalore, India. Three hundred and forty-eight cases of cancers of the oral cavity (excluding base tongue) were age and sex matched with controls from the same residential area but with no evidence of cancer. The relative risk due to pan tobacco chewing was elevated in both males and females, being appreciably higher in the latter (relative risk 25.3%; 95% confidence interval 11.2-57.3). A statistically significant (linear test for trend $P < 0.001$) dose response based on years, times per day and period of time chewed was seen. Any smoking (cigarette or bidi or both) had only slightly elevated risk of developing oral cancer, whereas a history of alcohol drinking or inhalation of snuff did not influence the risk. A new finding of our study was the markedly elevated risk of oral cancer in persons consuming ragi (*Eleusine coracana*, family graminae) in comparison to those not consuming ragi as staple cereal in their diet. There also appeared to be some interaction between ragi consumption and tobacco chewing with substantially higher relative risks in those who pursued both habits compared to those who gave a history of either.

Cancers of the oral cavity (ICD sites 140-141, 143-145) constitute one of the leading sites of cancer in men and women in India. The average annual age adjusted incidence rates in Bangalore for these combined sites of cancer is 5.0 per 100,000 in males and 9.3 per 100,000 in females (ICMR Annual Reports). Pan (consisting of betel leaf, areca nut and lime with or without tobacco) chewing is a fairly common social habit particularly in the older population, and the habit is relatively more frequently seen in women than men, as men more often smoke than chew tobacco.

There have been five previous case-control studies on oral cancer from this part of the world (Orr, 1933; Shanta & Krishnamoorthy, 1959, 1963; Hirayama, 1966; Sankarnarayanan *et al.*, 1989) but none of these is population based. This investigation attempts to consider in detail the effects of pan chewing, smoking, alcohol drinking and main dietary habits on the risk of developing oral cancer in the population of Bangalore.

### Subjects and methods

A population-based cancer registry was started at Kidwai Memorial Institute of Oncology, Bangalore from 1 January 1982 as part of the National Cancer Registry Programme of the Indian Council of Medical Research (ICMR, 1982-85; Bhargava & Nandakumar, 1987). The area covered is the resident (at least one year's residence) population of Bangalore Urban Agglomerate.

During the years 1982-84, 6,409 new cases (ICMR, 1985) of cancer were registered in the population based registry. Because of the wide range of diagnostic and therapeutic facilities offered, 73.4% (4,707 new cases) of these were seen at Kidwai Memorial Institute of Oncology at some point of time or the other (ICMR, 1982-85). The diagnostic or therapeutic status of the patient at the time of reference to the Institute varies. Some patients are referred on a mere symptom diagnosis or clinical suspicion of cancer by a general medical practitioner, others with a biopsy diagnosis and few others may have undergone surgery and referred for post operative radiotherapy and/or chemotherapy. However, when first seen, the vast majority of patients referred to the Institute await complete investigations and confirmation of diagnosis before subsequent treatment.

The cases initially chosen for the study were all registered cancers of ICD sites lip, tongue (excluding base of the tongue), alveolus, and mouth. During the period 1982-84 there were 399 cancers (133 males; 266 females) of these sites. Over 93% of them were microscopically confirmed.

Controls were chosen from among patients who attended Kidwai Memorial Institute of Oncology during the same time period, but who after investigations were proved not to have cancer. One control matched for sex, 5-year age group and area of residence (Bangalore Urban Agglomerate) as the corresponding case was selected. During the period 1982-84 there were 561 resident patients who attended Kidwai Memorial Institute of Oncology for diagnosis of ailments other than that of the oral cavity, but, who on investigation were found not to have any malignancy. Of the 561 subjects 471 were sex and age matched in order of registration to the 475 cases of oral cancer (including base tongue) that were registered during the same period. Four additional controls based on the same criteria were chosen from the first four 'proved non-cancers' of patients attending in 1985. Base tongue cancers (76 cases and their matched controls) were not included in the study.

Information on patients' habits (cases and controls) was sought by direct interview of the subjects by trained social investigators of the department of population based cancer registry. The items on which details were obtained included history of pan chewing with or without tobacco, the number of years since first started chewing, the number of times of chewing per day, as well as the period of time (in minutes) that the pan is retained in the mouth before being spat out or swallowed and whether the person retains the pan in the mouth during sleep. If a history of smoking was present further information on the habit included whether the person smoked bidi (a crude form of cigarette with less refined tobacco) or cigarette or both, the number of years since first started smoking and the actual number smoked per day. Similarly, a history of alcohol consumption included years since first started, frequency and type. Other items were with reference to inhalation of snuff (powdered tobacco), and details of food habits as to whether the subject solely depended on a vegetarian diet or not, the staple cereal consumed and the extent of spiciness of food.

Of the 399 cases details of the above mentioned habits were not available in 51 patients. This left 348 cases and 348 controls for the study.

Correspondence: A. Nandakumar.
Received 30 March 1990; and in revised form 11 June 1990.

Statistical analysis was by conditional logistic regression (Breslow & Day, 1980) which accounted for the matched design of the study and gave odds ratio estimates of relative risks (RR). Ninety-five per cent confidence intervals (CI) were calculated using the standard error of the regression estimates. Risk of one factor was adjusted for the risks of other factors. Those factors that were significant after adjustment of other factors were introduced stepwise into a multivariate model. Dose response was evaluated by tests for trend. Since only one female was a smoker and few females consumed alcohol, analysis for these factors was performed separately for males and females.

### Results

Table I shows the frequency of cases and controls. The average ages of cases and controls were nearly identical. There were slight differences between cases and controls in the proportion of different religions, language spoken and marital status. However, an appreciable difference was observed in the proportion of literates/illiterates among cases and controls. The proportion of literates among controls was more than twice that among cases.

Table II summarises the relative risks associated with smoking habit in males, including type, years and number smoked per day. Cigarette smoking and any smoking was associated with a slightly elevated relative risk and this remained significant after adjusting for the effect of pan tobacco chewing (RR 2.6; 95% CI 1.3–5.2; $P = 0.01$). The relative risk in chewers and smokers was not appreciably different from that in chewers alone. A dose response as indicated by statistically significant elevated relative risks in those persons who gave a history of smoking for more than 25 years, or of smoking more than ten cigarettes/bidis per day was observed. Our investigation showed that snuff inhalation and alcohol consumption in both males and females had minimal influence on the occurrence of oral cancer.

The number of cases and controls, the relative risk estimates and results of significance tests for pan chewing with and without tobacco are shown in Table III. The risk of oral cancer associated with pan tobacco chewing was significantly high in both males and females but the value was substantially higher in females. Pan chewing without tobacco did not increase the risk of oral cancer.

In calculating relative risks for dose–response parameters and history of chewing during sleep, subjects chewing pan without tobacco were considered as non-chewers. A dose response as indicated by increasing risk for years of chewing, number of times of chewing per day and period of retaining the pan in the mouth was observed (Table IV). A linear test for trend was statistically significant ($P < 0.001$) in all three instances. A history of keeping the pan in the mouth while asleep increased the relative risk two-fold.

Of the food habits that were considered, the main type of cereal consumed influenced the risk of oral cancer. A history of ragi or wheat as the main cereal consumed increased the relative risk several fold especially with respect to consumption of ragi. Subjects were dichotomised into never ragi and ever ragi consumption as the staple cereal (Table V).

Since the proportions of literates and illiterates among cases and controls differed the crude relative risk estimates for ever ragi consumption are shown separately for literates and illiterates with an overall adjusted (Mantel–Haenszel) relative risk as well (Table VI). The influence of educational

**Table I** Comparative features of cases and controls

| | *Cases* | *Controls* |
|---|---|---|
| Sex | | |
| Male | 115 | 115 |
| Female | 233 | 233 |
| Average age (years) | 54.8 | 55.2 |
| Religion | | |
| Hindu | 293 | 266 |
| Muslim | 33 | 52 |
| Christian | 20 | 27 |
| Others | 2 | 3 |
| Language spoken | | |
| Kannada | 110 | 122 |
| Tamil | 76 | 67 |
| Telugu | 96 | 72 |
| Urdu | 31 | 52 |
| Malayalam | 8 | 4 |
| Others | 19 | 31 |
| Marital status | | |
| Single | 11 | 11 |
| Married | 199 | 238 |
| Widowed | 126 | 96 |
| Divorced | 2 | 1 |
| Separated | 4 | 1 |
| Education | | |
| Illiterate | 261 (76.1%) | 144 (41.5%) |
| Literate | 82 (23.9%) | 203 (58.5%) |

**Table II** Relative risk (RR) estimates and results of significance tests of smoking habits in males

| | *Cases* | *Controls* | *RR* | *95% CI* | *P value* |
|---|---|---|---|---|---|
| Smoking | | | | | |
| No H/o smoking | 29 | 43 | 1.0 | – | – |
| Cigarette | 63 | 49 | 2.1 | 1.1– 4.2 | 0.03 |
| Bidi | 17 | 19 | 1.4 | 0.6– 3.0 | 0.41 |
| Cigarette + bidi | 6 | 4 | 2.3 | 0.6– 8.8 | 0.23 |
| No H/o smoking | 29 | 43 | 1.0 | – | – |
| Any smoking | 86 | 72 | 1.9 | 1.0– 3.4 | 0.04 |
| Smoke years | | | | | |
| No H/o smoking | 29 | 43 | 1.0 | – | – |
| 1–5 | 10 | 6 | 2.6 | 0.8– 8.6 | 0.12 |
| 6–15 | 9 | 14 | 0.9 | 0.3– 2.7 | 0.83 |
| 16–25 | 18 | 18 | 1.5 | 0.6– 3.5 | 0.39 |
| >25 | 49 | 34 | 2.2 | 1.1– 4.3 | 0.02 |
| Smoke (no. $day^{-1}$) | | | | | |
| No H/o smoking | 29 | 43 | 1.0 | – | – |
| 1–10 | 17 | 23 | 1.2 | 0.6– 2.7 | 0.63 |
| 11–20 | 37 | 24 | 2.5 | 1.2– 5.4 | 0.02 |
| >20 | 32 | 25 | 2.1 | 1.0– 4.4 | 0.06 |
| Chewing & smoking | | | | | |
| Neither | 14 | 38 | 1.0 | – | – |
| Chew only | 15 | 5 | 10.2 | 2.6–39.4 | <0.001 |
| Smoke only | 69 | 66 | 3.5 | 1.5– 8.2 | 0.003 |
| Chew + smoke | 17 | 6 | 9.2 | 2.6–32.2 | <0.001 |

CI = confidence interval. H/o = history of.

**Table III** Relative risk (RR) estimates and results of significance tests of chewing habits with and without tobacco

| | *Cases* | *Controls* | *RR* | *95% CI* | *P value* |
|---|---|---|---|---|---|
| Males | | | | | |
| No H/o chewing | 68 | 89 | 1.0 | – | – |
| Chewing without T | 15 | 15 | 1.5 | 0.6– 3.8 | 0.36 |
| Chewing with T | 32 | 11 | 4.0 | 1.8– 8.9 | <0.001 |
| No H/o chewing T | 83 | 104 | 1.0 | – | – |
| Tobacco chewers | 32 | 11 | 3.6 | 1.7– 7.9 | 0.001 |
| Females | | | | | |
| No H/o chewing | 19 | 144 | 1.0 | – | – |
| Chewing without T | 9 | 30 | 2.2 | 0.7– 6.5 | 0.17 |
| Chewing with T | 205 | 59 | 30.4 | 12.6–73.4 | <0.001 |
| No H/o chewing T | 28 | 174 | 1.0 | – | – |
| Tobacco chewers | 205 | 59 | 25.3 | 11.2–57.3 | <0.001 |
| Both sexes | | | | | |
| No H/o chewing | 87 | 233 | 1.0 | – | – |
| Chewing without T | 24 | 45 | 1.7 | 0.9– 3.5 | 0.114 |
| Chewing with T | 237 | 70 | 14.6 | 8.2–25.9 | <0.001 |
| No H/o chewing T | 111 | 278 | 1.0 | – | – |
| Tobacco chewers | 237 | 70 | 12.9 | 7.5–22.3 | <0.001 |

CI = confidence interval. H/o = history of. T = tobacco.

**Table IV** Relative risk (RR) estimates and results of significance tests of tobacco chewing habits with respect to duration of chewing (years), times per day, chewing period (in minutes) and chewing during sleep (both sexes)

| | *Cases* | *Controls* | *RR* | *95% CI* | *P value* |
|---|---|---|---|---|---|
| Chewing (years) | | | | | |
| No H/o chewing tobacco | 111 | 278 | 1.0 | – | – |
| 1–5 | 4 | 6 | 1.7 | 0.3– 9.3 | 0.539 |
| 6–15 | 23 | 7 | 10.3 | 3.6–29.6 | <0.001 |
| 16–25 | 56 | 20 | 12.4 | 5.6–27.2 | <0.001 |
| >25 | 154 | 37 | 15.95 | 8.4–30.2 | <0.001 |
| Chewing (times per day) | | | | | |
| No H/o chewing tobacco | 111 | 278 | 1.0 | – | – |
| 1–4 | 82 | 33 | 9.3 | 4.9–17.5 | <0.001 |
| 5–9 | 98 | 28 | 12.8 | 6.6–25.0 | <0.001 |
| ⩾10 | 35 | 8 | 16.6 | 6.3–44.3 | <0.001 |
| Chewing period (minutes) | | | | | |
| No H/o chewing tobacco | 111 | 278 | 1.0 | – | – |
| ⩽5 | 5 | 3 | 6.4 | 0.9–45.1 | 0.063 |
| 6–10 | 67 | 20 | 9.7 | 4.7–19.8 | <0.001 |
| 11–20 | 59 | 13 | 16.5 | 7.2–37.4 | <0.001 |
| 21–30 | 54 | 17 | 13.2 | 5.8–30.0 | <0.001 |
| >30 | 11 | 6 | 6.6 | 1.6–27.0 | 0.008 |
| Chewing during sleep | | | | | |
| No H/o chewing tobacco | 111 | 278 | 1.0 | – | – |
| No H/o chewing during sleep | 108 | 47 | 8.5 | 4.7–15.2 | <0.001 |
| H/o chewing during sleep | 103 | 19 | 17.7 | 8.7–36.1 | <0.001 |

CI = confidence interval. H/o = history of.

**Table V** Frequency, relative risk (RR) estimates and results of significance tests of main cereal consumed (both sexes)

| *Main Cereal* | *Cases* | *Controls* | *RR* | *95% CI* | *P value* |
|---|---|---|---|---|---|
| Rice | 187 | 337 | 1.0 | – | – |
| Ragi | 143 | 6 | 29.3 | 11.9 – 72.3 | <0.001 |
| Jowar | 1 | 2 | 3.6 | 0.1 – 95.4 | 0.445 |
| Wheat | 15 | 1 | 15.0 | 1.98–113.6 | 0.009 |
| Rice | 187 | 337 | 1.0 | – | – |
| Ragi | 143 | 6 | 31.2 | 12.6 – 77.4 | <0.001 |
| Other | 16 | 3 | 10.4 | 2.3 – 46.3 | 0.002 |
| No ragi | 203 | 340 | 1.0 | – | – |
| Ragi | 143 | 6 | 28.40 | 11.6 – 69.3 | <0.001 |

CI = confidence interval.

status was further observed by introducing, stepwise, the variables ever ragi consumption, educational status and history of pan tobacco chewing, into a conditional logistic regression model (Table VII). The risk of ever ragi consumption remained elevated after adjusting for pan tobacco chewing and educational status (RR 27.4; 95% CI 9.9–75.9; $P<0.001$).

In order to determine whether there was an interaction between pan tobacco chewing and consumption of ragi as the main cereal, the relative risk in subjects who chewed tobacco as well as consumed ragi was estimated and a marked increase in relative risk (RR 242.6; 95% CI 52.6–1119) was seen, compared to those who chewed tobacco without consuming ragi (RR 12.5; 95% CI 6.3–24.9) or those who consumed ragi without chewing tobacco (RR 32.5; 95% CI 8.8–119.5). Although the estimated risk in a multiplicative model would be $12.5 \times 32.5 = 406.25$ for significant interaction the estimated risk of 242.6 is very high (Table VIII).

**Table VI** Estimates of crude and adjusted (Mantel & Haenszel) relative risk (RR) and results of significance tests of ever ragi consumption as main cereal and educational status

| | Cases | Controls | RR (crude) | 95% CI | P value |
|---|---|---|---|---|---|
| *Literates* | | | | | |
| No ragi | 53 | 198 | 1.0 | – | – |
| Ragi | 29 | 3 | 36.11 | 11.3–180.6 | <0.001 |
| *Illiterates* | | | | | |
| No ragi | 147 | 141 | 1.0 | | – |
| Ragi | 113 | 3 | 36.13 | 11.3–180.7 | <0.001 |
| | | | *RR (adjusted)* | | |
| *Literates & illiterates* | | | | | |
| No ragi | 200 | 339 | 1.0 | – | – |
| Ragi | 142 | 6 | 36.12 | 15.8– 90.3 | <0.001 |

The educational status in 4 cases and 1 control was unknown. CI = confidence interval.

**Table VII** Relative risk (RR) estimates and results of significance tests of ever ragi consumed, educational status and history of pan tobacco chewing in a stepwise model

| | RR | 95% CI | P value |
|---|---|---|---|
| *Ragi & educational status* | | | |
| Ragi as main cereal | 26.7 | 10.6–67.5 | <0.001 |
| (0 = no ragi, 1 = ragi | | | |
| Educational status | 5.3 | 3.1– 8.9 | <0.001 |
| (0 = literate, 1 = illiterate) | | | |
| *Ragi & pan tobacco chewing* | | | |
| Ragi as main cereal | 26.3 | 9.8–70.9 | <0.001 |
| Pan tobacco chewing | 11.9 | 6.2–22.8 | <0.001 |
| (0 = no tobacco, 1 = tobacco) | | | |
| *Ragi, educational status & pan tobacco chewing* | | | |
| Ragi as main cereal | 27.4 | 9.9–75.9 | <0.001 |
| Educational status | 3.1 | 1.7– 5.9 | <0.001 |
| Pan tobacco chewing | 8.9 | 4.5–17.3 | <0.001 |

CI = confidence interval.

**Table VIII** Relative risk (RR) estimates and confidence intervals (CI) of tobacco chewing and ragi consumption habits (both sexes)

| | | *Tobacco chewing* | |
|---|---|---|---|
| *Ragi consumption* | | *No* | *Yes* |
| No | RR | 1.00 | 12.5 |
| | (95% CI) | – | (6.3–24.9) |
| | Cases/cont. | 76/272 | 127/68 |
| Yes | RR | 32.5 | 242.6 |
| | (95% CI) | (8.8–119.5) | (52.6–1119.0) |
| | Cases/cont. | 35/4 | 108/2 |

### Discussion

This study confirmed reports of previous investigators (Ellis, 1921; Davidson, 1923; Jussawalla & Deshpande, 1971; IARC, 1985) that pan tobacco chewing is a major risk factor in the occurrence of cancers of the oral cavity. Further, a dose response as measured by chewing years, chewing times per day, period of time chewed and retention of chewing quid overnight while asleep could be clearly demonstrated. In males presence of a history of any smoking was associated with a significantly elevated relative risk. An unexpected new finding of this study, however, was the increased risk when ragi was the staple cereal consumed. This elevated risk was not influenced by any of the other known risk factors and remained unchanged even after stratification and adjusting for educational status, which was thought to be a possible confounder because of differing proportions of literates and illiterates among cases and controls.

Alcohol consumption or snuff inhalation did not emerge as independent risk factors in our study, nor did they enhance or interact with pan tobacco chewing or staple cereal consumed.

The relationship between tobacco either chewed or smoked and development of cancer of the oral cavity is known (Ellis, 1921; Orr, 1933). However, a distinction of anatomic subsites in relating risk factors appears important. By way of embryologic and anatomic development, and also because in pan chewing the anterior tongue and other areas of the mouth are exposed to a greater degree than the base of the tongue, it appears necessary to distinguish this portion of the tongue from the rest of the oral cavity. Our analysis on the risk associated with base tongue cancers is being reported separately.

A statistically significant dose response with respect to chewing habits in this study suggests that certain modifications in chewing habits could substantially reduce the risk of developing oral cancer. The most important of these and perhaps the easiest to follow by the average chewer would be to spit out the pan as early as possible (within 5 min) and not to retain the quid in the mouth overnight while asleep.

Smoking in this study did emerge as an independent risk factor although the strength of the association was greater for pan tobacco chewing. A dose response with smoking could be elicited, but appeared weak. Bidi smoking has been shown to be an independent risk factor for oral cancer by earlier investigators (Sanghvi, 1955; Jussawalla & Deshpande, 1971). We did not find any notable difference in relative risks between bidi and cigarette smokers or in those who smoked both.

Although the extensive study by the IARC (1988) has shown an elevated risk of oral cancers in those who consumed alcohol, our study, like the preceding one (Sankarnarayanan *et al.*, 1989) from this region, did not show any association whatsoever. Any slight elevations in risk were lost once this factor was adjusted for pan tobacco chewing and/or smoking.

An indication of a possible protective effect of dietary factors, like milk, milk products and fish, on the risk of oral cancer has been reported earlier (Notani & Sanghvi, 1976). However, it is for the first time that any relationship of oral cancer to a staple cereal consumed is being suggested. Since questions on diet for this study were asked routinely and not for testing any hypothesis related to diet, the finding here of highly elevated relative risk of oral cancer when ragi (*Eleusine coracina*; family graminae) was the staple cereal consumed calls for a more detailed assessment of diet and nutritional status in future studies on oral cancer. It is possible that our finding could be confounded by these and other various known and unknown risk factors. Some of these could be in relation to oral hygiene, socioeconomic status and other dietary habits of those consuming rice in contrast to ragi or wheat as the main cereal. Nonetheless, the finding here of substantially elevated risk in ragi consumers is important, particularly because of the marked increase in risk when combined with pan tobacco chewing.

That over 73% or resident cancer patients are referred to

Kidwai Memorial Institute of Oncology (ICMR, 1982–85) makes data collection on a population basis through direct patient interviews relatively easy. An added advantage is the almost total absence of any problem related to confidentiality. The questioning and recording of details of patient habits by the social investigators was done immediately after the patient arrived at the institute and before any clinical examination or investigations. Therefore, the social investigators were not aware of the diagnosis or whether the patient was proved as cancer or not at the time of the interview and any interviewer bias is unlikely. The main limitation of this study is that only one control per case was used and that detailed information on socioeconomic and educational status was not obtained.

In conclusion our study confirmed the role of pan tobacco chewing, and also demonstrated a significant dose response on the risk of oral cancer, but dietary factors, in particular ragi consumption, appear to enhance that risk considerably.

This study was possible because of the population-based cancer registry commenced by the Indian Council of Medical Research as part of the network of National Cancer Registries in India and the authors gratefully acknowledge the encouragement and funding given by the Council and Dr Usha K. Luthra, Programme Director, National Cancer Registry Programme and Additional Director General, Indian Council of Medical Research. Many thanks are due to Dr Bruce K. Armstrong, Commissioner of Health, Western Australia, for his constructive comments on the earlier drafts of the paper.

### References

BHARGAVA, M.K. & NANDAKUMAR, A. (1987). In *Cancer Incidence in Five Continents Vol. V*, Muir, C.S., Waterhouse, J.A.H., Mack, T., Powell, J. & Whelan, S. (eds) p. 408. IARC: Lyon.

BRESLOW, N.E. & DAY, N.E. (1980). *Statistical Methods in Cancer Research*. IARC: Lyon.

DAVIDSON, J. (1923). Betel chewing and cancer (correspondence). *Br. Med. J.*, **ii**, 733.

ELLIS, A.G. (1921). Betel nut chewing and its effects, including cancer of the mouth. *Arch. Intern. Med.*, **28**, 252.

HIRAYAMA, T. (1966). An epidemiologic study of oral and pharyngeal cancer in Central and South east Asia. *Bull. WHO*, **34**, 41.

IARC (1985). Evaluation of the carcinogenic risk of chemicals to humans: tobacco habits other than smoking; betel quid and arecanut chewing; and some related nitrosamines. *IARC Monogr.*, **37**, 109.

IARC (1988). Evaluation of the carcinogenic risk to humans; alcohol drinking. *IARC monogr.*, **44**, 167.

ICMR (1982–1985). *Annual Reports of National Cancer Registry Project of India (NCRP)*. Indian Council of Medical Research: New Delhi.

JUSSAWALLA, D.J. & DESHPANDE, V.A. (1971). Evaluation of cancer risk in tobacco chewers and smokers: an epidemiologic assessment. *Cancer*, **28**, 244.

NOTANI, P.N. & SANGHVI, L.D. (1976). Role of diet in the cancers of the oral cavity. *Ind. J. Cancer*, **13**, 156.

ORR, I.M. (1933). Oral cancer in betel nut chewers in Travancore. Its aetiology, pathology and treatment. *Lancet*, **ii**, 575.

SANGHVI, L.D., RAO, K.C.M. & KHANOLKAR, V.R. (1955). Smoking and chewing of tobacco in relation to cancer of upper alimentary tract. *Br. Med. J.*, **i**, 1111.

SANKARNARAYANAN, R., DUFFY, S.W., DAY, N.E. & 2 others (1989). A case control investigation of cancer of the oral tongue and the floor of the mouth in Southern India. *Int. J. Cancer*, **44**, 617.

SHANTHA, V. & KRISHNAMOORTHY, S. (1963). A study of aetiology of carcinomas of the upper alimentary tract. *Br. J. Cancer*, **17**, 8.